# Acute myocardial infarction and coronary vasospasm associated with the ingestion of cayenne pepper pills in a 25-year-old male

**DOI:** 10.1186/1865-1380-5-5

**Published:** 2012-01-20

**Authors:** Ozgur Sogut, Halil Kaya, Mehmet Tahir Gokdemir, Yusuf Sezen

**Affiliations:** 1 Department of Emergency Medicine, Harran University, Sanliurfa, Turkey; 2 Department of Cardiology, Harran University, Sanliurfa, Turkey

## Abstract

Capsaicin, one of the major active components of cayenne pepper pills, is an over-the-counter substance with sympathomimetic activity used commonly by young individuals for weight loss. Here we report the case of a previously healthy young male who developed severe chest pain after using cayenne pepper pills for slimming and sustained an extensive inferior myocardial infarction. Electrocardiography combined with a bedside transthoracic echocardiogram confirmed the diagnosis of acute myocardial infarction. The patient denied using illicit substances, and he had no risk factors for coronary artery disease. His medication history revealed that he had recently started taking cayenne pepper pills for slimming. A subsequent coronary angiogram revealed patent coronary arteries, suggesting that the mechanism was vasospasm. We postulate that the patient developed acute coronary vasospasm and a myocardial infarction in the presence of this known sympathomimetic agent. This case highlights the potential danger of capsaicin, even when used by otherwise healthy individuals.

## Background

For slimming, the use of weight loss pills flavored with Mexican pepper seeds is common among females. Originally called "La Jiao Shou Shen," cayenne (chili) pepper pills have been imported to Turkey from the Far East. China and Russia are the only countries in the world where chili pepper pills are sold. Capsules containing 400 and 600 mg of chili pepper are sold for weight loss; patches and gels are used for pain relief and slimming, respectively [1].

Cayenne pepper pills contain pungent ingredients called capsaicinoids, and one of the active components of cayenne pepper is capsaicin [2]. Capsaicin accelerates energy expenditure and suppresses body fat accumulation by activating the sympathetic nervous system (SNS) in animals and humans [3-6]. However, this substance is associated with cardiotoxicity, including coronary vasospasm, supraventricular tachycardia, and acute atrial fibrillation [1,7]. Capsaicin also prolongs the cardiac action potential in atrial and ventricular myocytes, an effect that is associated with the inhibition of potassium currents [8,9]. Coronary vasospasm and acute myocardial infarction (AMI) associated with the use of topical capsaicin patches to relieve back pain have been reported recently [7]. Here, we describe the first reported case of coronary vasospasm and AMI suspected of being caused by the use of cayenne pepper pills for slimming.

## Case presentation

**Figure 1:**
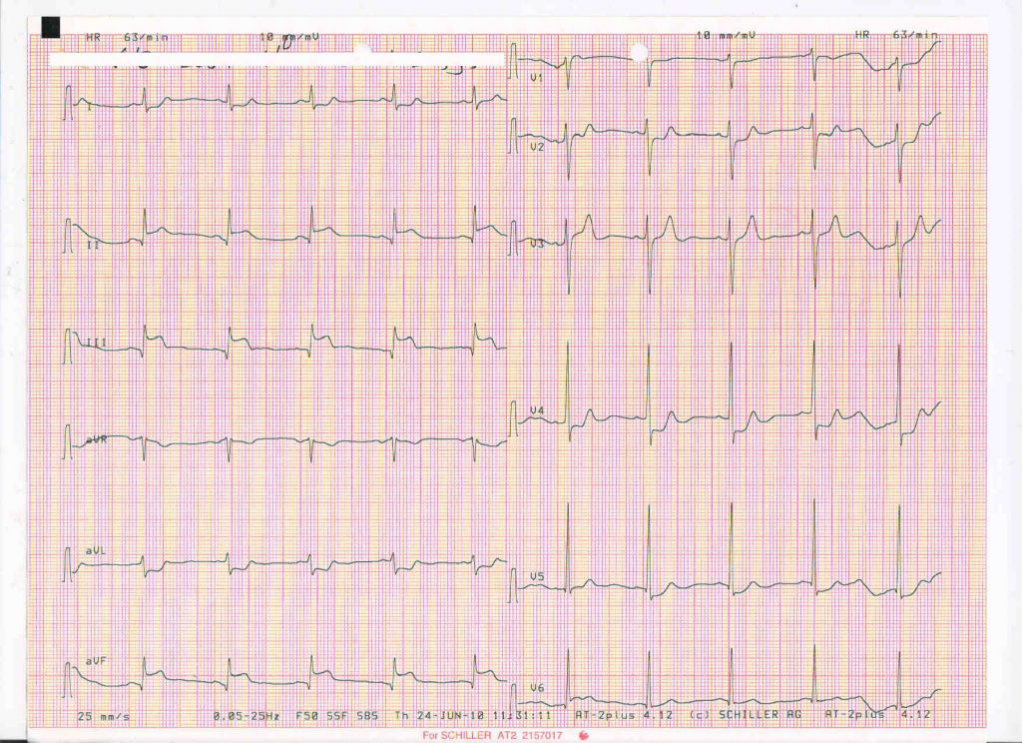
**The patient's initial ECG, showing an acute inferior myocardial infarction**.

A 25-year-old male was admitted to our emergency department complaining of severe chest pain 2 h after its onset. The pain radiated to the ulnar aspect of the patient's left arm, neck, and jaw. An electrocardiogram (ECG) demonstrated an ST-segment elevation of 7 mm in leads II, III, and aVF, consistent with an inferior wall AMI (Figure 1). A bedside transthoracic echocardiogram showed a non-dilated left ventricle with inferior hypokinesia, confirming a recent myocardial infarction. On physical examination, the patient was conscious but extremely anxious. His blood pressure was 90/55 mmHg, pulse rate 62 beats/min, and respiratory rate 25 breaths/min. The patient had no cardiac risk factors for coronary artery disease, no history of recent emotional or physical stress, and had not ingested any illicit substances.

**Figure 2:**
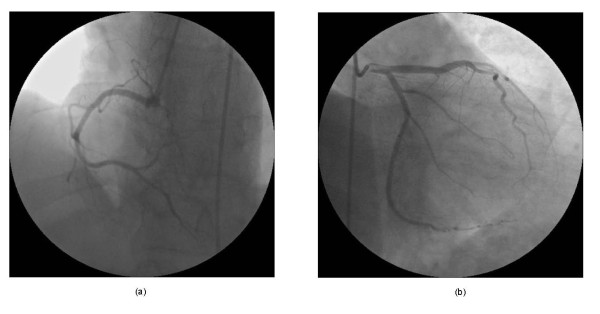
**Coronary angiography showed patent (**a**) left and (**b**) right coronary artery systems**.

The patient began taking oral cayenne pepper pills for slimming, once a day, 5 days before presenting to the hospital. The patient's cardiac enzyme levels on admission were normal, but subsequently showed a crescendo-decrescendo pattern with a peak troponin T value of 4.6 ng/ml (normal < 0.01 ng/ml). The patient was diagnosed with an inferior AMI, and appropriate treatment was instituted, including controlled intravenous fluids with normal saline, aspirin, sublingual nitroglycerin, low-dose morphine, and unfractionated heparin. The patient's pain and ECG changes disappeared after the administration of sublingual nitroglycerin. An urgent diagnostic coronary angiogram within 3 h of the onset of chest pain revealed normal right and left coronary artery systems (Figure 2). Since the coronary arteries were normal, oral diltiazem (60 mg) three times daily was added to his therapy. One month later, the patient had experienced no angina attacks, and echocardiography showed mild hypokinesia of the inferior wall.

## Discussion

Chili peppers are a major source of natural capsaicinoids, including capsaicin, dihydrocapsaicin, nordihydrocapsaicin, homodihydrocapsaicin, and homocapsaicin [10]. Capsaicin is the active component of chili peppers and has multiple pharmacological and physiological effects, including analgesic, anticancer, anti-inflammatory, antioxidant, and anti-obesity effects; moreover, it promotes the rapid elimination of toxins and fats generated in the body [5,10]. The characteristic effects of capsaicinoids may be of therapeutic value for pain relief, cancer prevention, and weight loss [7,10]. In addition to these therapeutic effects, capsaicinoids have various side effects on the cardiovascular system [1,7]. In animal experiments, capsaicinoids accelerated catecholamine secretion by activating the adrenal sympathetic efferent nerve [4,11]. Capsaicinoids act on the cardiovascular system largely as potential agonists of capsaicin receptor or transient receptor potential vanilloid subfamily member 1 (TRPV1) [10]. Hachiya et al. [12] reported that capsaicin enhances adrenal medullary adrenaline secretion in humans, which transiently elevates the blood pressure and heart rate. In isolated working rat hearts, Szolcsányi et al. [13] found that capsaicin elicited concentration-dependent constriction of the coronary arteries and decreased coronary flow by acting on VR1 capsaicin receptors. They suggested that capsaicin-induced coronary artery spasm in the rat heart was mediated by endothelin release from sensory nerve terminals, resulting in deteriorated cardiac function. In our case, we attributed the occurrence of vasospasm and AMI to the use of cayenne pepper pills for weight loss, which might have evoked a coronary artery spasm by eliciting neural endothelin release and catecholamine secretion, indicating marked sympathomimetic activity as a result of its intense stimulatory effect on the SNS.

Myocardial infarction in teenagers and young adults is uncommon, and the underlying mechanism is related mainly to congenital coronary anomalies, coagulopathy, premature atherosclerosis, coronary artery spasm, or drugs [14]. Most commonly used stimulants (e.g., cocaine, cigarettes, marijuana, alcohol, butane, and amphetamines) have been reported to cause AMI secondary to intense coronary vasospasm in youths when used alone or in combination [14,15]. These substances exaggerate the vasospastic activity of other drugs, enhance endothelial dysfunction, increase platelet aggregation, increase sympathetic activity, and decrease the myocardial oxygen supply [14]. In our case, there was no history of substance abuse. In addition, the patient had no cardiac risk factors, and his chest pain began after using oral capsaicin for slimming for 5 days. The patient sustained an extensive inferior myocardial infarction.

Coronary artery spasm in association with myocardial infarction primarily affects younger individuals with or without underlying atherosclerotic lesions [14,15]. However, a substantial number of cases have angiographically normal coronary arteries [15-18]. Myocardial infarction with normal coronary arteries is an uncommon phenomenon resulting from numerous conditions, but the clinical presentation is similar to that of myocardial infarction with coronary atherosclerosis. Cigarette smokers and cocaine users are more prone to developing this condition [17]. The possible mechanisms of myocardial infarction in individuals with normal coronary arteries are a hypercoagulable state, coronary embolism, an imbalance between oxygen demand and supply, intense sympathetic stimulation, non-atherosclerotic coronary disease, coronary trauma, coronary vasospasm, coronary thrombosis, and endothelial dysfunction [14,17]. It has been recommended that once normal coronary arteries are identified on subsequent angiography, calcium channel blockers should be added to the treatment regimen, since coronary vasospasm seems to play a major role in the pathophysiology of this condition [17].

The majority of cases with clinical and electrocardiographic signs of acute coronary events have been diagnosed based on the patency of coronary vessels seen in an elective coronary angiogram [16-18]. Since the provocation of coronary artery spasm following AMI posses a high risk, we did not perform a provocation test in our case. After coronary angiography showing no occlusive lesions, our patient was treated with the calcium antagonist diltiazem. The observed improvement in his clinical and electrocardiographic findings and the relief of his chest pain after stopping the cayenne pepper pills support the role of capsaicin in the development of coronary vasospasm through increased sympathetic stimulation.

## Conclusions

This is the first report linking the use of cayenne pepper pills for slimming to an acute coronary syndrome, and one of the few cases associated with capsaicin. Capsaicin, a sympathomimetic agent, may be implicated in the initiation of coronary vasospasm and acute myocardial infarction in the absence of substance abuse, particularly in previously healthy teenagers and young adults. We suggest that this product, which may increase the risk of life-threatening cardiovascular events, be closely supervised and controlled by relevant institutions worldwide.

### Consent

The authors obtained permission from the patient to display the images and photographs in scientific journals.

## Competing interests

The authors declare that they have no competing interests.

## Authors' contributions

OS and YS treated the patient and wrote the case report. HK and MTG critically revised the manuscript. All authors read and approved the final manuscript.

## Authors' information

Ozgur Sogut, Halil Kaya, and Mehmet Tahir Gokdemir are at Harran University, School of Medicine, Department of Emergency Medicine, Sanliurfa, Turkey. Yusuf Sezen is at Harran University, School of Medicine, Department of Cardiology, Sanliurfa, Turkey.
